# Collagen gene cluster expression and liver fibrogenesis in patients with biliary atresia: a preliminary study

**DOI:** 10.1186/s13104-023-06636-0

**Published:** 2023-12-01

**Authors:** Gunadi, Dyah Ayu Puspitarani, Khanza Adzkia Vujira, Fadila Dyah Trie Utami, Edita Mayda Devana, Fiqih Vidiantoro Halim, Kristy Iskandar, Akhmad Makhmudi

**Affiliations:** 1 Pediatric Surgery Division, Department of Surgery/Genetics Working Group/Translational Research Unit, Faculty of Medicine, Public Health and Nursing, Universitas Gadjah Mada/Dr.Sardjito Hospital, Yogyakarta, 55281 Indonesia; 2 Department of Child Health/Genetics Working Group, Faculty of Medicine, Public Health and Nursing, Universitas Gadjah Mada/UGM Academic Hospital, Yogyakarta, 55281 Indonesia

**Keywords:** Biliary atresia, Collagen gene cluster expressions, Developing country, Kasai procedure, Liver fibrogenesis

## Abstract

**Objective:**

Biliary atresia (BA) is a progressive fibro-obliterative disease of the biliary tract, which results in end-stage liver disease. However, liver fibrosis progression may continue even after Kasai surgery. Recent evidence showed that collagen plays a pivotal role in the progression of liver fibrosis in BA. However, most studies were conducted in developed countries. We investigated the expressions of the collagen gene cluster (*COL6A1, COL6A2, COL6A3*, and *COL1A1*) in BA patients in Indonesia.

**Results:**

There was a significant down-regulated expression of *COL6A1* (ΔC_T_ 9.06 ± 2.64 *vs.* 5.42 ± 2.41; *p* = 0.0009), *COL6A2* (ΔC_T_ 8.25 ± 2.07 *vs.* 5.77 ± 3.51; *p* = 0.02), *COL6A3* (ΔC_T_ 11.2 ± 6.08 *vs.* 6.78 ± 3.51; *p* = 0.024), and *COL1A1* (ΔC_T_ 3.26 ± 1.71 *vs.* 0.19 ± 2.76; *p* = 0.0015) in BA patients compared to controls. Interestingly, the collagen gene cluster expressions were significantly associated with the presence of cirrhosis (*p* = 0.0085, 0.04, and 0.0283 for *COL6A1, COL6A2*, and *COL6A3*, respectively). In conclusion, our study shows the changes in the collagen gene cluster, particularly collagen type I and VI, expressions in patients with BA in a particular developing country. Our findings suggest the role of these collagen gene clusters in the liver fibrogenesis of BA.

**Supplementary Information:**

The online version contains supplementary material available at 10.1186/s13104-023-06636-0.

## Introduction

Biliary atresia (BA) is the most common cause of cholestasis in infants under three months. It is characterized by microinflammation and fibrosis of intra- and extrahepatic bile ducts [1]. If therapy is not given, children suffering from BA will have progressive liver fibrosis and cirrhosis and usually will not survive more than two years of age [2].

Liver fibrosis in BA might manifest rapidly after Kasai surgery. Understanding liver fibrosis in BA might benefit patient prognosis, development of biomarkers for diagnosis, and targeted therapy of liver fibrosis in BA patients [3]. A recent study showed that the expression of the collagen gene cluster was strongly associated with liver fibrosis, including BA patients [4–7]. However, most studies were conducted in developed countries [5–7]. Therefore, our study investigated the role of *COL6A1, COL6A2, COL6A3*, and *COL1A1* expressions on liver fibrosis in BA patients in Indonesia.

## Material and methods

### Patients

Twenty liver tissues of BA patients were acquired during Kasai surgery. The type of BA was determined using the Kasai classification system [8]. Cirrhosis was determined as bridging fibrosis with > 50% of portal tracts encompassed and nodular architecture [9]. At the same time, 18 control liver specimens were obtained from patients who underwent surgeries or biopsies for other diseases, including intrahepatic cholestasis (n = 7), choledochal cyst (5), internal bleeding (1), intraabdominal tumor (n = 2), gastric volvulus (n = 1), liver abscess (1), and Alagille syndrome (n = 1).

### Total RNA isolation

Total RNA was isolated from 25 to 30 mg of liver tissue using the Quick-RNA Miniprep Kit (Zymo Research, Irvine, California, US). The RNA concentration was quantified by a NanoDrop 2000 Spectrophotometer (Thermo Scientific, Wilmington, DE, USA) with the OD260/280 ratios ranging from 1.8 to 2.0 to ensure RNA purity. The samples were immediately stored at − 80 °C for future use.

### Quantitative RT-PCR

One-step qPCR was performed using SensiFAST SYBR No-ROX Kit (Bioline, Tennessee, USA) and BioRad CFX Real-Time PCR System (California, USA) for collagen gene cluster (*COL6A1, COL6A2, COL6A3,* and *COL1A1*). A housekeeping gene, *GAPDH*, was used as a reference gene. The reaction mix contained 10 μL sensiFAST one-step mix, 0.8 μL of each primer, 0.2 μL Reverse Transcriptase, 0.4 μL RNase Inhibitor, RNAse free water was added to reach 16 μL. Lastly, the mRNA was added, resulting in the final volume of 20 μL. PCR cycling conditions were: 45 °C for 10 min followed by 1 cycle of 95 °C for 2 min and 40 cycles of 95 °C for 5 s (denaturation), 60 °C for 1 min (Annealing/Extension). The primers that were used for collagen gene cluster were as follows: *COL6A1* 5′—TAAAGGCTACCGAGGCGATG—3′ (forward) and 5′—GCCGTCTTCTCCCCTTTCAC—3′ (reverse); *COL6A2*: 5′—CTCCTCGGGACCAGGACTTC—3′ (forward) and 5′—CGGTCTTCTCTGGGCAGTTG—3′ (reverse); *COL6A3*: 5′—TTAGCCAGCACTCGCTATCC—3′ (forward) and 5′—TTACTGGGGCCGATGTTGAG—3′ (reverse); *COL1A1*: 5′—CAATGCTGCCCTTTCTGCTCCTTT—3′ (forward) and 5′—ATTGCCTTTGATTGCTGGGCAGAC—3′ (reverse) [4]; and *GAPDH*: 5′—GCACCGTCAAGGCTGAGAAC—3′ (forward) and 5′—TGGTGAAGACGCCAGTGGA—3′ (reverse) [10].

### Statistical analysis

The expression of *COL6A1, COL6A2, COL6A3,* and *COL1A1* were determined using the Livak method (2^−ΔΔCT^). The expression of *COL6A1, COL6A2, COL6A3,* and *COL1A1* was presented as mean ± standard deviation (SD). The normality of the continuous variables was defined by the Kolmogorov–Smirnov test. The differences in the expressions of collagen gene clusters between BA patients and controls were analyzed using an independent t-test with a significance value of *p* < 0.05. All statistical analyses were performed using the IBM Statistical Package for the Social Sciences (SPSS) version 21 (Chicago, USA).

## Results

### Baseline characteristics

We ascertained 20 BA patients and 18 controls. Most BA patients were female (60%) and type 3 (60%). The median age of patients who underwent the Kasai procedure was 124 (IQR, 95.5–174.5) days (Table 1).

**Table 1:** Characteristics of BA patients after the Kasai procedure in our institution

Characteristics	n (%); median (IQR)
Gender	
Male	8 (40)
Female	12 (60)
Type of biliary atresia	
1	1 (5)
2A	6 (30)
2B	1 (5)
3	12 (60)
Age at Kasai surgery (days)	124 (95.5–174.5)
Laboratory findings before Kasai surgery	
Total bilirubin (mg/dL)	11.36 (9.69–14.52)
Direct bilirubin (mg/dL)	9.59 (6.68–12.38)
Aspartate aminotransferase (U/L)	192 (131–333)
Alanine aminotransferase (U/L)	119 (103–232)
Gamma-glutamyl transferase (U/L)	716 (239–1117)
Alkaline phosphatase (U/L)	520 (337–687.5)
Laboratory findings after Kasai surgery	
Total bilirubin (mg/dL)	11.32 (6.53–14.62)
Direct bilirubin (mg/dL)	9.01 (5.7–11.33)
Aspartate aminotransferase (U/L)	164 (75–215)
Alanine aminotransferase (U/L)	114.5 (68–151.75)
Gamma-glutamyl transferase (U/L)	527 (313–758.25)
Alkaline phosphatase (U/L)	282 (100–324)
Liver biopsy	
Cirrhosis	5 (25)
Non-cirrhosis	15 (75)
Outcomes	
Survived	7 (35)
Died	13 (65)

*IQR* interquartile range

### Expression of the collagen gene cluster in BA patients

There was a significant down-regulated expression of *COL6A1* (*p* = 0.0009), *COL6A2* (*p* = 0.02), *COL6A3* (*p* = 0.024), and *COL1A1* (*p* = 0.0015) in liver BA patients compared to controls with the fold change of 12.53-, 5.58-, 21.35-, and 8.41-times, respectively (Additional file 1: Fig. S1; Table 2).

**Table 2:** Expression of the collagen gene cluster in BA patients and control liver

	Biliary atresia (ΔC_T_ ± SD)	Control (ΔC_T_ ± SD)	ΔΔC_T_ (95% CI)	Fold change (2^−ΔΔC^_T_)	*p*-value
*COL6A1*	9.06 ± 2.65	5.42 ± 2.41	3.65 (1.65–5.65)	12.53	0.0009*
*COL6A2*	8.25 ± 2.07	5.77 ± 3.51	2.48 (0.43–4.53)	5.58	0.02*
*COL6A3*	11.2 ± 6.08	6.78 ± 3.51	4.42 (0.63–8.21)	21.35	0.024*
*COL1A1*	3.26 ± 1.71	0.19 ± 2.76	3.07 (1.29–4.86)	8.41	0.0015*

^*^p < 0.05 is considered statistically significant

### Association between collagen gene cluster expression and outcomes

Interestingly, the collagen gene cluster expressions were significantly associated with the presence of cirrhosis (*p* = 0.0085, 0.04, and 0.0283 for *COL6A1, COL6A2,* and *COL6A3*, respectively) but not with the survival of BA patients and age at Kasai procedure (Table 3).

**Table 3:** Association of collagen gene cluster expression and outcomes of BA patients after Kasai procedure

	*COL6A1*	*COL6A2*	*COL6A3*	*COL1A1*
Cirrhosis (ΔC_T_ ± SD) (n = 5)	12.71 ± 4.02	9.76 ± 4.2	17.29 ± 9.44	2.46 ± 1.45
Non-cirrhosis (ΔC_T_ ± SD) (n = 15)	7.51 ± 1.65	6.51 ± 2.18	9.74 ± 3.76	2.35 ± 0.84
ΔΔC_T_ (95% CI)	5.2 (1.65–9.74)	3.26 (0.01–6.5)	7.55 (0.92–14.17)	0.11 (− 1.52–1.74)
Fold change **(2**^**−ΔΔC**^_**T**_**)**	36.63	9.55	187.04	1.08
*p*-value	0.0085*	0.04*	0.028*	0.879
Age at Kasai surgery ≥ 90 days (n = 15)	10.34 ± 7.51	8.41 ± 2.9	12.2 ± 7.35	3.5 ± 1.78
Age at Kasai surgery < 90 days (n = 5)	10.25 ± 3.02	7.34 ± 4.82	10.85 ± 1.81	2.61 ± 1.16
ΔΔC_T_ (95% CI)	0.09 (-7.31 – 7.48)	1.07 (− 2.64 – 4.79)	1.35 (− 7.97–10.67)	2.9 (− 1.17–2.95)
Fold change **(2**^**−ΔΔC**^_**T**_**)**	1.06	2.11	2.55	1.85
*p*-value	0.981	0.551	0.762	0.371
Died (ΔC_T_ ± SD) (n = 13)	11.02 ± 4.68	8.66 ± 3.27	11.99 ± 4.84	3.4 ± 1.2
Survived (ΔC_T_ ± SD) (n = 7)	9.1 ± 1.99	8.35 ± 2.42	8.27 ± 4.47	2.53 ± 1.94
ΔΔC_T_ (95% CI)	1.92 (− 2.07–5.91)	0.31 (− 2.75–3.37)	3.72 (− 1.76–9.21)	0.87 (− 1.33–3.07)
Fold change **(2**^**−ΔΔC**^_**T**_**)**	3.78	1.24	13.21	1.83
*p*-value	0.323	0.832	0.168	0.409

^*^p < 0.05 was considered statistically significant

## Discussion

Our study shows the aberrant expressions of collagen type VI genes (*COL6A1, COL6A2,* and *COL6A3)* in BA patients compared with controls. A previous study showed that the collagen gene cluster (*COL6A1, COL6A2, COL6A3*, and *COL1A1)* expressions were associated with the hepatic stellate cells (HSCs) activation [4]. *Collagen VI (COL6A1, COL6A2*, and *COL6A3*) genes, but not *COL1A1*, have been associated with liver fibrosis [11–13]. The accumulation of type VI collagen might cause the destruction of the liver's structure and function in liver fibrosis [11]. It is compatible with our findings that *COL6A1, COL6A2*, and *COL6A3* expressions*,* not *COL1A1,* were strongly associated with the presence of cirrhosis (Table 3). Moreover, *collagen type VI* has been shown as a potent activating factor for HSCs and an inducing factor for fibrosis-associated gene expression changes, including *αSMA* and *TGF-β* [12].

In addition, CO6-MMP, a collagen type VI fragment, was elevated in bile-duct ligation (BDL) and the carbon tetrachloride (CCl4) induced fibrosis rat models. It suggests its role in liver fibrogenesis [4, 11]. However, previous studies investigated the association of *collagen type VI* genes with liver fibrosis in other diseases, including cholestatic liver diseases and acute liver injury, not BA, as the novelty of our study [4, 11–13]. In addition, previous studies showed the aberrant other collagen gene expressions (*COL10A1, COL15A1, COL22A1*, *COL9A2* [5] and *COL3A1*, *COL1A2* [7]), not *COL6A1, COL6A2, COL6A3*, and *COL1A1* genes as our study, in patients with BA. These differences are another novelty of our findings.

Our study also shows the aberrant expressions of *COL1A1* in BA patients compared to the control. A recent study demonstrated the involvement of *COL1A1* in liver fibrosis through *miRNA-29* signaling [14]. Moreover, another study showed a constantly high expression of 12 genes, including the *collagen* gene cluster, in BA patients one year after a successful Kasai surgery compared to at the time of the Kasai procedure [5]. This suggests that the fibrosis process evolved with time instead of as a one-time process. The genes involved may not be a consequence but a precursor of the fibrotic changes [5]. Moreover, Baiocchini et al*.* [15] also identified *COL1A1* as one of the molecules associated with liver fibrosis in HCV-infected patients.

Interestingly, our findings show downregulated collagen gene cluster expressions in cirrhosis patients compared with non-cirrhosis patients (Table 3). However, a previous study revealed a significantly higher expression of the *collagen* gene cluster in the fibrosis group than in the inflammation group [16]. Our previous study showed that the expression of *COL1A1* and *COL6A1* were significantly downregulated in the hypospadias patients with more severe chordee than the milder one (Indonesian) [17]. In contrast, there is no difference in collagen intensity between hypospadias patients and controls (Caucasian) [18]. Moreover, some collagen genes have different molecular evolutionary characteristics among different populations [19].

## Conclusions

Our study shows the changes in the collagen gene cluster, particularly *collagen type I* and *VI* genes, expressions in patients with BA in a particular developing country. Our findings suggest the role of this *collagen* gene cluster in the liver fibrogenesis of BA.

## Limitations

Our study has several limitations, including a small sample size and one pediatric surgical center that might not reflect the Indonesian population. Moreover, we do not validate the protein levels of the *collagen *gene cluster in BA, i.e., lack of pathological images and collagen deposition staining of the liver tissues in patients with BA due to limited resources. Notably, the control liver specimens were patients with chronic liver and gallbladder diseases, not subjects with normal liver tissue. These facts should be considered during the interpretations of our findings. In this study, we focus on the effect of the collagen gene cluster expressions in liver fibrogenesis of BA, while the association between the prognostic factors, including histopathological findings, with the survival rate of BA patients after Kasai procedures have been reported in our previous studies [9, 20].

### Supplementary Information

**Additional file 1**. Box-plot graph of ΔCT value of the collagen gene cluster expressions in liver BA patients and controls. Box-plot graph of ΔCT value reveals the mean values as lines across the box.

## Data Availability

All data generated or analyzed during this study are included in the submission. The raw data are available from the corresponding author upon reasonable request.

## Abbreviations

BA: Biliary atresia
IQR: Interquartile range
