# Supplementary figures and images for: Collagen gene cluster expression and liver fibrogenesis in patients with biliary atresia: a preliminary study
Source: BMC Res Notes. 2023 Dec 1;16:356. doi: 10.1186/s13104-023-06636-0 (PMC10690962; doi:10.1186/s13104-023-06636-0)

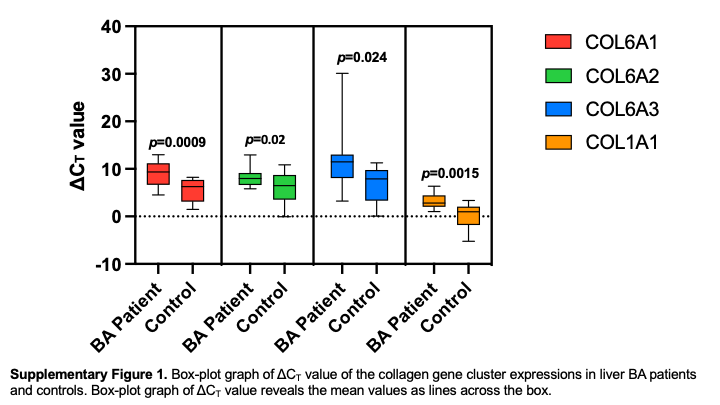

Supplement: Supplementary file 1 — Additional file 1. Box-plot graph of ΔCT value of the collagen gene cluster expressions in liver BA patients and controls. Box-plot graph of ΔCT value reveals the mean values as lines across the box. [file 13104_2023_6636_MOESM1_ESM.tiff]
